# Effective endoscopic treatment of intrahepatic stones after Roux-en-Y hepaticojejunostomy: a pediatric case

**DOI:** 10.1055/a-2098-1626

**Published:** 2023-06-15

**Authors:** Koichi Soga, Shun Takakura, Junki Yumoto

**Affiliations:** Department of Gastroenterology, Omihachiman Community Medical Center, Shiga, Japan

An 11-year-old Japanese boy presented to our hospital with intrahepatic biliary duct stones and regional cholangitis. He had undergone cholecystectomy, complete excision of the extrahepatic biliary duct, and Roux-en-Y hepaticojejunostomy for choledochal cysts (Todani classification IVa) at the age of 8 years. Abdominal magnetic resonance imaging confirmed cystic dilatation of the left hepatic duct and the presence of left intrahepatic biliary duct stones ( Fig. 1 a ).

**Fig. 1:**
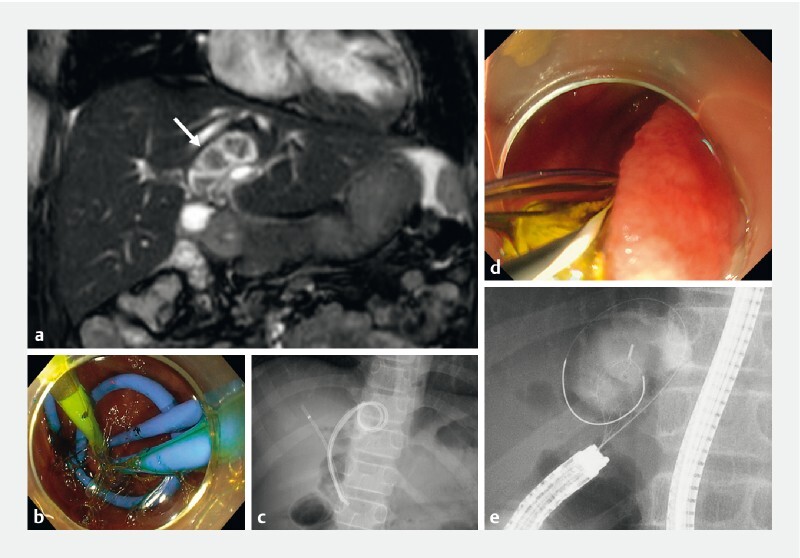
Images of endoscopic treatment of intrahepatic bile duct stones showing: **a** cystic dilatation of the left hepatic duct and intrahepatic duct stones (arrow) on abdominal magnetic resonance imaging; **b, c** endoscopic biliary drainage using balloon enteroscopy with the placement of multiple plastic stents seen on: **b** endoscopy; **c** fluoroscopy; **d, e** removal of the intrahepatic bile duct stones, which was performed 3 months later.

During long school vacations, three separate endoscopic procedures using balloon enteroscopy (SIF-190 or H290S; Olympus, Japan) were performed to improve the long-term prognosis following the choledochal cyst surgery. During the first session, endoscopic biliary drainage with multiple plastic stents was performed to reduce the size of the stones by abrasion between the plastic stents and the stones, while also avoiding recurrence of the cholangitis ( Fig. 1 b, c ). The second session 3 months later involved removal of the left intrahepatic biliary duct stones using a basket catheter (8-wire Nitinol basket; Medico's Hirata Inc, Osaka, Japan) ( Fig. 1 d, e ) and placement of a fully covered self-expandable metal stent (FCSEMS; Bonastent, Sewoon Medical Inc., Seoul, South Korea) at the left intrahepatic duct to dilate the anastomosis of the left hepatic duct ( Fig. 2 a, b ). The third endoscopic procedure 6 months later aimed to confirm the stones had disappeared and to remove the FCSEMS and plastic stent. The intrahepatic biliary stones were confirmed to have disappeared following these procedures, and the patient’s outcome was good during 12 months of follow-up ( Fig. 2 c–e ; Video 1 ).

**Fig. 2:**
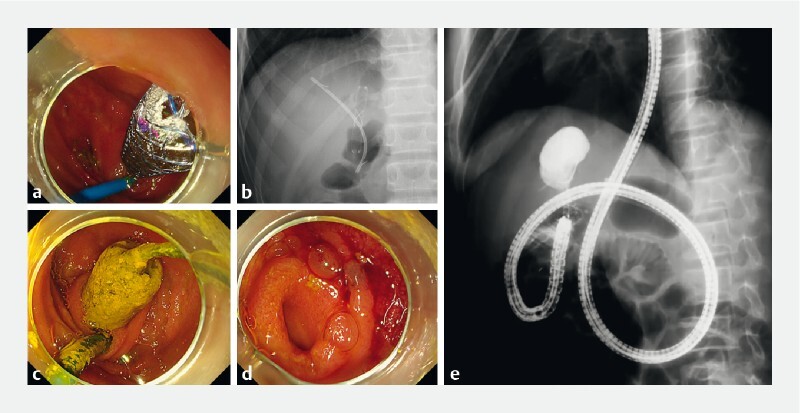
Images of the treatment of the biliojejunal anastomosis following intrahepatic bile duct stone extraction showing: **a, b** a fully covered self-expandable metal stent (FCSEMS) in position to dilate the anastomosis and help removal of residual intrahepatic biliary stones; **c** after confirmation of stone drainage 6 months later, a snare used to remove the FCSEMS during balloon enteroscopy; **d** confirmation of the dilation of the biliojejunal anastomosis after FCSEMS removal; **e** no obvious residual intrahepatic biliary stones on cholangiography.

**Video 1** Three separate endoscopic procedures were performed using balloon enteroscopy to improve the long-term prognosis after choledochal cyst surgery.


Postoperative complications of choledochal cysts generally worsen the outcome and represent a major challenge 1 . Patients with choledochal cysts are mainly operated on during childhood, and a patient’s quality of life is rapidly reduced through the appearance of symptoms. These scheduled endoscopic procedures can be considered an option to the standard procedure for choledochal cyst anastomotic strictures, allowing for treatment planning that corresponds to the lifestyle of the affected child and allows the anastomosis to be extended for a period of time with an FCSEMS.

Endoscopy_UCTN_Code_TTT_1AR_2AH

## Notes

Endoscopy E-Videos

https://eref.thieme.de/e-videos
*E-Videos* is an open access online section of the journal *Endoscopy* , reporting on interesting cases and new techniques in gastroenterological endoscopy. All papers include a high-quality video and are published with a Creative Commons CC-BY license. Endoscopy E-Videos qualify for HINARI discounts and waivers and eligibility is automatically checked during the submission process. We grant 100% waivers to articles whose corresponding authors are based in Group A countries and 50% waivers to those who are based in Group B countries as classified by Research4Life (see: https://www.research4life.org/access/eligibility/ ). This section has its own submission website at https://mc.manuscriptcentral.com/e-videos
